# Supplementary material for: Hypoxia Inducible Factor 1A Supports a Pro-Fibrotic Phenotype Loop in Idiopathic Pulmonary Fibrosis
Source: Int J Mol Sci. 2021 Mar 24;22(7):3331. doi: 10.3390/ijms22073331 (PMC8078165; doi:10.3390/ijms22073331)
Supplement: Supplementary file 1 [file ijms-22-03331-s001.zip › supp Table 1.docx]

| **Company** | **Dilution** | **Source** | **Cat. No** | **Target** |
| --- | --- | --- | --- | --- |
| Abcam | 1:200 (IHC)  1:1000 (WB) | Rabbit | Ab2185 | HIF1α |
| Santa Cruz | 1:250 (IHC)  1:500 (WB) | Mouse mAb | Sc-21734 | TIMP1 |
| Abcam | 1:500 (IHC)  1:2000 (WB) | Rabbit | Ab66705 | PAI-1 |
| Abcam | 1:5000 (WB) | Mouse mAb | mAbcam9484 | GAPDH |
| Cell Signaling Technologies | 1:1000 (WB) | Rabbit mAb | #8457 | Beta-Actin |
| Millipore | 1:5000 (WB) | Goat | #AP308P | Peroxidase conjugated anti-mouse |
| Millipore | 1:5000 (WB) | Goat | #AP132P | Peroxidase conjugated anti-Rabbit |

Supplementary Table 1: Antibodies used in the study
