# Supplementary material for: Hypoxia Inducible Factor 1A Supports a Pro-Fibrotic Phenotype Loop in Idiopathic Pulmonary Fibrosis
Source: Int J Mol Sci. 2021 Mar 24;22(7):3331. doi: 10.3390/ijms22073331 (PMC8078165; doi:10.3390/ijms22073331)
Supplement: Supplementary file 1 [file ijms-22-03331-s001.zip › supp Table 2-primers.docx]

|  | **Forward (5'-3')** | **Reverse (5'-3')** |
| --- | --- | --- |
| SERPINE1 (PAI-1) | CAGACCAAGAGCCTCTCCAC | GGTTCCATCACTTGGCCCAT |
| VEGFA | AAGGCCAGCACATAGGAGAGATGA | TCTTTCTTTGGTCTGCATTCACA |
| TIMP1 | CTTCTGGCATCCTGTTGTTG | GGTATAAGGTGGTCTGGTTG |
| HIF1A | GAAAAAGATAAGTTCTGAACGTCGAAA | ATGTGGAAGTGGCAACTGATGA |
| ACTA2 | TGAGAAGAGTTACGAGTTGCCTGAT | GCAGACTCCATCCCGATGAA |
| COL1a | CGAAGACATCCCACCAATCAC | CAGATCACGTCATCGCACAAC |
| HPRT1 | CCTCATGGACTAATTATGGACAGGA | GCACACAGAGGGCTACAATGTG |
| GAPDH | CTCTGCTCCTCCTGTTCGAC | TTAAAAGCAGCCCTGGTGAC |
| ACTB | GACCACACCTTCTACAATGAG | GCATACCCCTCGTAGATGGG |

Supplementary Table 2: List of primers
